# Supplementary material for: The genome sequence of the Club-Horned Wood Borer Wasp, Trypoxylon clavicerum Lepeletier de Saint Fargeau & Audinet-Serville, 1828 (Hymenoptera: Crabronidae)
Source: Wellcome Open Res. 2026 Apr 22;11:240. [Version 1] doi: 10.12688/wellcomeopenres.26358.1 (PMC13237539; doi:10.12688/wellcomeopenres.26358.1)
Supplement: Peer review history

# Reviewer report: Reviewer response for version 1

**DOI:** 10.21956/wellcomeopenres.29029.r155524

**Published:** 2026-06-04

**Authors:** Sara E Miller

**Affiliations:** 1 University of Missouri at Saint Louis, St Louis, Missouri, USA

The authors present a new high quality nuclear and mitochondrial genome assembly for the solitary Club-Horned Wood Borer wasp, *Trypoxylon clavicerum *(Hymenoptera: Crabronidae).

 Genome sequencing and assembly were completed using standard Darwin Tree of Life Consortium protocols from a single specimen and scaffolded with Hi-C data from a second specimen. The resulting genome has a length of 270.99 Mb, scaffolded into 9 chromosomes, and is contiguous and high quality with a scaffold N50 of 24.81 Mb and 94.9% coverage of BUSCO genes. No annotation is provided. The final assembly exceed Earth BioGenome Project Assembly Standards benchmarks.

Are sufficient details of methods and materials provided to allow replication by others?

Yes

Is the rationale for creating the dataset(s) clearly described?

Yes

Are the datasets clearly presented in a useable and accessible format?

Yes

Are the protocols appropriate and is the work technically sound?

Yes

Reviewer Expertise:

Hymenoptera genetics and genomics, evolutionary ecology

I confirm that I have read this submission and believe that I have an appropriate level of expertise to confirm that it is of an acceptable scientific standard.

# Reviewer report: Reviewer response for version 1

**DOI:** 10.21956/wellcomeopenres.29029.r155526

**Published:** 2026-06-03

**Authors:** Fatih Dikmen

**Affiliations:** 1 Istanbul University, Istanbul, Turkey

The Club-Horned Wood Borer Wasp, *Trypoxylon clavicerum*, is a solitary wasp, widespread throughout the Palaearctic and a small bee species belonging to Apoidea. Sequencing the genome of this species may provide an evolutionary baseline for comparative bee genomics, serving as a critical phylogenetic bridge to decode the origins of the clade Anthophila. Because Crabronidae represents one fo the the closest living relatives to modern bees within the superfamily Apoidea, the *de novo* assembly of this species' genome offers a powerful outgroup to trace the profound metabolic and morphological shifts required to transition from a carnivorous, hunting lifestyle to an obligate pollen- and nectar-based herbivory. Furthermore, as an exclusively solitary organism, its genetic architecture serves as a vital reference point for uncovering the molecular roots of eusociality, allowing researchers to isolate the specific gene duplications and regulatory networks that drove the evolution of complex social behaviors in modern bees.

Are sufficient details of methods and materials provided to allow replication by others?

Yes

Is the rationale for creating the dataset(s) clearly described?

Yes

Are the datasets clearly presented in a useable and accessible format?

Yes

Are the protocols appropriate and is the work technically sound?

Yes

Reviewer Expertise:

Bee taxonomy, genomics, zoology.

I confirm that I have read this submission and believe that I have an appropriate level of expertise to confirm that it is of an acceptable scientific standard.
